# Supplementary material for: Kinetic analysis of hemoglobin detergency by probability density functional method
Source: PLoS One. 2020 Aug 7;15(8):e0237255. doi: 10.1371/journal.pone.0237255 (PMC7413519; doi:10.1371/journal.pone.0237255)
Supplement: Supplementary file 1 [file pone.0237255.s001.docx]

[30°C]						
Before			R		K/S	
	F	B	F	B	F	B
1	42.1	45.21	0.421	0.4521	0.398148	0.332
2	42.78	46.95	0.4278	0.4695	0.38267	0.299713
3	42.63	45.58	0.4263	0.4558	0.386033	0.324872
4	41.3	44.39	0.413	0.4439	0.417154	0.34833
5	44.02	46.91	0.4402	0.4691	0.355947	0.300421
6	44	47.62	0.44	0.4762	0.356364	0.288079
7	43.65	46.35	0.4365	0.4635	0.363725	0.310499
8	43.27	46.29	0.4327	0.4629	0.371885	0.311597
9	43.66	46.79	0.4366	0.4679	0.363513	0.302554
10	43.46	47.14	0.4346	0.4714	0.367783	0.29637
11	43.39	45.2	0.4339	0.452	0.369289	0.332195
12	42.46	46.63	0.4246	0.4663	0.389879	0.305421
13	42.78	47.56	0.4278	0.4756	0.38267	0.289104
14	45.99	45.4	0.4599	0.454	0.317143	0.328322
15	47.34	43.64	0.4734	0.4364	0.292889	0.363938
16	43.32	47.36	0.4332	0.4736	0.370801	0.292543
17	45.52	48.23	0.4552	0.4823	0.326018	0.277849
18	45.1	47.55	0.451	0.4755	0.334147	0.289275
19	42.25	47.32	0.4225	0.4732	0.394682	0.293236
20	45.61	47.33	0.4561	0.4733	0.324301	0.293062

1-wash			R		K/S		removal(%)		
	F	B	F	B	F	B	F	B	mean
1	44.91	48.07	0.4491	0.4807	0.337888	0.2805	15.59189	16.0768	15.66
2	45.71	49.03	0.4571	0.4903	0.322403	0.264934	16.24431	12.07385	
3	45.91	48.56	0.4591	0.4856	0.318637	0.272454	18.00232	16.73578	
4	44.16	46.65	0.4416	0.4665	0.353046	0.305061	15.80975	12.85194	
5	46.97	49.86	0.4697	0.4986	0.299359	0.252108	16.43635	16.7312	
6	46.91	50.11	0.4691	0.5011	0.300421	0.248355	16.22931	14.37106	16.10
7	46.95	49.28	0.4695	0.4928	0.299713	0.26101	18.1821	16.56026	
8	46.36	49.55	0.4636	0.4955	0.310316	0.256832	17.09189	18.25897	
9	46.17	49.3	0.4617	0.493	0.313804	0.260699	14.12774	14.38865	
10	46.7	49.55	0.467	0.4955	0.304164	0.256832	17.86449	13.88737	
11	46.75	48.52	0.4675	0.4852	0.303269	0.273103	18.46067	18.43545	20.75
12	47.13	51.18	0.4713	0.5118	0.296545	0.232844	24.67719	24.70622	
13	45.76	50.28	0.4576	0.5028	0.321457	0.245831	16.49908	15.5969	
14	50.14	48.61	0.5014	0.4861	0.247908	0.271645	22.66422	17.89824	
15	51.3	48.69	0.513	0.4869	0.231159	0.270355	21.9503	26.56518	
16	50.35	53.05	0.5035	0.5305	0.244799	0.207757	35.08453	30.18569	26.98
17	49.63	51.67	0.4963	0.5167	0.255605	0.22603	22.39908	19.46731	
18	49.31	51.19	0.4931	0.5119	0.260543	0.232703	22.82402	20.37777	
19	49.65	54.41	0.4965	0.5441	0.255299	0.190999	36.39035	36.30902	
20	50.69	50.85	0.5069	0.5085	0.239838	0.237534	27.01608	19.73277	

2-wash			R		K/S		removal(%)		
	F	B	F	B	F	B	F	B	mean
1	46.24	49.05	0.4624	0.4905	0.312515	0.264618	22.15689	21.03461	20.20
2	46.46	49.73	0.4646	0.4973	0.308495	0.254079	19.993	15.8421	
3	46.68	48.88	0.4668	0.4888	0.304523	0.267313	21.77258	18.37709	
4	45.47	48.01	0.4547	0.4801	0.326976	0.2815	22.23903	19.85041	
5	47.91	50.41	0.4791	0.5041	0.283173	0.243917	21.13761	19.56787	
6	47.27	51.3	0.4727	0.513	0.294103	0.231159	18.06204	20.59206	20.56
7	47.82	50.38	0.4782	0.5038	0.284688	0.244357	22.44981	22.13288	
8	47.58	50.7	0.4758	0.507	0.288762	0.239693	23.07546	23.97301	
9	47.07	49.58	0.4707	0.4958	0.297598	0.256371	18.73382	15.87636	
10	47.47	50.53	0.4747	0.5053	0.290647	0.242161	21.6601	19.04019	
11	48.69	50.91	0.4869	0.5091	0.270355	0.236675	27.66403	29.80012	29.61
12	48.79	52.76	0.4879	0.5276	0.26875	0.211488	32.02619	31.97626	
13	47.48	52.34	0.4748	0.5234	0.290475	0.216992	24.84991	25.99144	
14	51.61	50.97	0.5161	0.5097	0.226854	0.235819	29.55607	29.21186	
15	53.43	50.16	0.5343	0.5016	0.202954	0.24761	31.97953	33.02167	
16	53.25	55.71	0.5325	0.5571	0.205217	0.176055	46.10573	41.47232	38.25
17	52.35	54.5	0.5235	0.545	0.21686	0.189931	34.72434	33.02852	
18	51.14	53.46	0.5114	0.5346	0.233408	0.202579	31.23829	31.22902	
19	52.63	56.91	0.5263	0.5691	0.213179	0.16313	47.38737	46.20641	
20	53.29	53.62	0.5329	0.5362	0.204712	0.200588	38.25122	32.86217	

3-wash			R		K/S		removal(%)		
	F	B	F	B	F	B	F	B	mean
1	46.76	49.79	0.4676	0.4979	0.30309	0.253168	24.5955	24.60904	23.75
2	47.46	50.64	0.4746	0.5064	0.290819	0.240562	24.75725	20.53485	
3	47.66	50.11	0.4766	0.5011	0.287398	0.248355	26.34685	24.43001	
4	45.63	48.5	0.4563	0.485	0.32392	0.273428	22.99263	22.24798	
5	48.59	51.01	0.4859	0.5101	0.271968	0.23525	24.39222	22.56924	
6	48.57	52.01	0.4857	0.5201	0.272292	0.221404	24.38961	24.12124	24.91
7	48.76	51.21	0.4876	0.5121	0.269231	0.232422	26.84019	26.12686	
8	48.55	51.08	0.4855	0.5108	0.272616	0.234257	27.55756	25.78559	
9	47.83	49.97	0.4783	0.4997	0.284519	0.25045	22.45093	17.91174	
10	48.66	52.09	0.4866	0.5209	0.270838	0.220327	27.22246	26.70908	
11	49.53	51.57	0.4953	0.5157	0.257139	0.227406	31.35941	32.69198	35.40
12	50.01	53.51	0.5001	0.5351	0.24985	0.201955	37.02335	35.22138	
13	48.72	53.67	0.4872	0.5367	0.269873	0.199969	30.40298	32.12719	
14	53	52.27	0.53	0.5227	0.208396	0.217922	35.59842	34.86383	
15	55.85	52.58	0.5585	0.5258	0.174505	0.213832	42.09541	42.61024	
16	55.08	57.68	0.5508	0.5768	0.183171	0.155252	52.24445	48.87875	44.08
17	55.03	56.72	0.5503	0.5672	0.183745	0.165123	45.25839	42.34822	
18	53.73	53.13	0.5373	0.5313	0.199229	0.206738	41.83702	29.73083	
19	53.67	57.39	0.5367	0.5739	0.199969	0.158182	50.83609	47.96377	
20	54.77	54.76	0.5477	0.5476	0.186759	0.186875	43.99388	37.73515	

4-wash			R		K/S		removal		
	F	B	F	B	F	B	F	B	mean
1	46.94	50.26	0.4694	0.5026	0.29989	0.246127	25.42357	26.80696	26.50
2	47.93	51.07	0.4793	0.5107	0.282838	0.234398	26.90835	22.67454	
3	48.51	50.93	0.4851	0.5093	0.273265	0.23639	30.12183	28.25018	
4	46.73	48.99	0.4673	0.4899	0.303626	0.265566	27.99738	24.58303	
5	49.05	51.66	0.4905	0.5166	0.264618	0.226167	26.52716	25.71481	
6	49.47	52.48	0.4947	0.5248	0.258064	0.215144	28.51737	26.38582	28.32
7	49.35	51.84	0.4935	0.5184	0.259921	0.223706	29.48444	29.04337	
8	48.74	52.17	0.4874	0.5217	0.269551	0.219255	28.40832	30.78716	
9	48.53	51.55	0.4853	0.5155	0.272941	0.227682	25.74165	25.73876	
10	49.35	52.54	0.4935	0.5254	0.259921	0.214356	30.28793	28.8064	
11	50.42	52.46	0.5042	0.5246	0.24377	0.215407	35.09771	36.43537	38.99
12	50.96	54.32	0.5096	0.5432	0.235962	0.192071	40.6954	38.58586	
13	49.55	54.62	0.4955	0.5462	0.256832	0.188516	33.91794	36.25546	
14	53.75	53.04	0.5375	0.5304	0.198983	0.207885	38.68	38.03343	
15	56.81	53.65	0.5681	0.5365	0.164177	0.200216	45.76802	46.47523	
16	56.23	58.7	0.5623	0.587	0.170355	0.145289	55.81283	52.42574	49.04
17	55.87	57.4	0.5587	0.574	0.174285	0.15808	48.26791	44.99415	
18	54.37	55.69	0.5437	0.5569	0.191475	0.176277	44.24147	40.70315	
19	55.29	59.34	0.5529	0.5934	0.180773	0.139302	55.84796	54.66891	
20	56.39	56.24	0.5639	0.5624	0.168632	0.170247	49.79176	43.64426	

[40°C]						
Before			R		K/S	
	F	B	F	B	F	B
21	44.04	46.9	0.4404	0.469	0.355532	0.300598
22	40.23	45.74	0.4023	0.4574	0.444004	0.321835
23	43.77	47.32	0.4377	0.4732	0.361185	0.293236
24	44.49	46.22	0.4449	0.4622	0.346298	0.312883
25	44.27	48.51	0.4427	0.4851	0.350783	0.273265
26	42.88	47.23	0.4288	0.4723	0.380445	0.294799
27	41.6	47.16	0.416	0.4716	0.409923	0.296021
28	44.25	46.89	0.4425	0.4689	0.351194	0.300775
29	46.05	44.76	0.4605	0.4476	0.316026	0.340869
30	43.9	48.42	0.439	0.4842	0.358452	0.274731
31	42.86	46.52	0.4286	0.4652	0.380889	0.307407
32	43.36	47.93	0.4336	0.4793	0.369937	0.282838
33	46.12	45.94	0.4612	0.4594	0.314728	0.318076
34	43.22	45.55	0.4322	0.4555	0.372972	0.325445
35	42.47	45.85	0.4247	0.4585	0.389652	0.319763
36	42.89	46.61	0.4289	0.4661	0.380223	0.305781
37	42.8	46.63	0.428	0.4663	0.382224	0.305421
38	43.4	45.98	0.434	0.4598	0.369074	0.317329
39	43.21	47.39	0.4321	0.4739	0.37319	0.292025
40	42.75	46.36	0.4275	0.4636	0.383341	0.310316

1-wash			R		K/S		removal(%)		
	F	B	F	B	F	B	F	B	mean
21	47.25	50.06	0.4725	0.5006	0.294451	0.249101	17.76264	17.8228	19.98
22	45.27	50.55	0.4527	0.5055	0.330834	0.24187	26.17587	25.78083	
23	47.43	50.27	0.4743	0.5027	0.291335	0.245979	19.98428	16.78301	
24	48.27	49.52	0.4827	0.4952	0.27719	0.257293	20.65164	18.45476	
25	47.53	51.72	0.4753	0.5172	0.289617	0.225344	18.03654	18.31825	
26	49.18	51.17	0.4918	0.5117	0.262573	0.232985	31.9622	21.8318	28.99
27	50.34	54	0.5034	0.54	0.244946	0.195926	41.42429	35.20004	
28	51.17	53.67	0.5117	0.5367	0.232985	0.199969	34.81508	34.86728	
29	49.88	48.83	0.4988	0.4883	0.251806	0.268111	21.09984	22.10099	
30	48.81	52.08	0.4881	0.5208	0.26843	0.220461	25.95855	20.62938	
31	52.8	55.07	0.528	0.5507	0.21097	0.183285	46.02016	41.96894	35.64
32	48.33	51.49	0.4833	0.5149	0.276204	0.228512	26.16212	20.03329	
33	51.44	50.87	0.5144	0.5087	0.229206	0.237248	28.21887	26.37879	
34	52.99	53.74	0.5299	0.5374	0.208524	0.199106	45.51418	40.26315	
35	51.77	53.5	0.5177	0.535	0.22466	0.202079	43.6496	38.19623	
36	54.5	57.31	0.545	0.5731	0.189931	0.158998	51.63089	49.90585	42.72
37	49.2	52.54	0.492	0.5254	0.26226	0.214356	32.37347	30.99987	
38	50.63	51.97	0.5063	0.5197	0.240707	0.221944	35.91562	31.20567	
39	52.98	55.37	0.5298	0.5537	0.208652	0.179866	45.51156	40.00476	
40	56.17	57.87	0.5617	0.5787	0.171005	0.153356	57.12873	52.55581	

2-wash			R		K/S		removal(%)		
	F	B	F	B	F	B	F	B	mean
21	48.62	51.35	0.4862	0.5135	0.271483	0.23046	24.44181	24.27459	26.93
22	47.09	52.34	0.4709	0.5234	0.297247	0.216992	33.94463	33.80129	
23	49.03	50.68	0.4903	0.5068	0.264934	0.239982	27.53779	18.91265	
24	50.03	50.85	0.5003	0.5085	0.24955	0.237534	28.91125	25.01434	
25	49.25	53.28	0.4925	0.5328	0.261478	0.204838	26.33407	26.15665	
26	51.03	53.95	0.5103	0.5395	0.234966	0.196534	39.44833	34.70573	38.71
27	52.23	56.68	0.5223	0.5668	0.218454	0.165545	48.07611	45.8839	
28	53.31	55.67	0.5331	0.5567	0.20446	0.1765	43.21626	42.98495	
29	52	51.15	0.52	0.5115	0.221538	0.233267	31.04425	32.68506	
30	51.13	54.68	0.5113	0.5468	0.233549	0.187811	36.01671	33.04065	
31	55.34	57.96	0.5534	0.5796	0.180206	0.152464	54.35218	52.39055	46.44
32	51.63	53.94	0.5163	0.5394	0.226579	0.196656	40.01317	31.78078	
33	54.11	53.51	0.5411	0.5351	0.194594	0.201955	39.63964	37.89677	
34	55.82	56.52	0.5582	0.5652	0.174836	0.167243	54.838	50.41761	
35	54.71	56.53	0.5471	0.5653	0.18746	0.167136	53.49128	49.53773	
36	58.64	60.56	0.5864	0.6056	0.14586	0.128427	63.58843	60.29978	56.77
37	54	56.26	0.54	0.5626	0.195926	0.170031	50.2744	46.08872	
38	54.86	56.18	0.5486	0.5618	0.185711	0.170896	51.30286	47.90584	
39	57.32	59.22	0.5732	0.5922	0.158896	0.140409	59.27437	54.07816	
40	60.99	62.15	0.6099	0.6215	0.124757	0.115255	69.57181	65.31311	

3-wash			R		K/S		removal(%)		
	F	B	F	B	F	B	F	B	mean
21	49.35	51.42	0.4935	0.5142	0.259921	0.229484	27.80417	24.61222	31.66
22	48.51	52.92	0.4851	0.5292	0.273265	0.209422	39.49145	36.24184	
23	49.91	53.15	0.4991	0.5315	0.251353	0.206484	31.42324	30.80957	
24	50.92	52.32	0.5092	0.5232	0.236532	0.217257	32.80141	31.74583	
25	50.18	54.34	0.5018	0.5434	0.247313	0.191832	30.51119	31.12826	
26	53.24	56.18	0.5324	0.5618	0.205344	0.170896	47.48077	43.76054	45.31
27	54.48	57.89	0.5448	0.5789	0.190168	0.153157	55.17854	50.24048	
28	55.86	57.08	0.5586	0.5708	0.174395	0.161364	52.07122	48.22034	
29	53.51	52.7	0.5351	0.527	0.201955	0.212267	37.47852	39.06416	
30	52.58	55.81	0.5258	0.5581	0.213832	0.174947	41.70243	37.93073	
31	57.82	59.5	0.5782	0.595	0.153853	0.137836	61.48948	57.33662	52.93
32	53.71	56.35	0.5371	0.5635	0.199475	0.169061	47.57826	41.95659	
33	55.92	55.32	0.5592	0.5532	0.173734	0.180432	46.52232	44.92075	
34	57.92	58.59	0.5792	0.5859	0.15286	0.146338	60.92047	57.07974	
35	56.43	57.53	0.5643	0.5753	0.168204	0.156762	58.58564	52.90492	
36	61.02	62.75	0.6102	0.6275	0.124503	0.110563	69.38307	66.37374	61.98
37	56.32	57.99	0.5632	0.5799	0.169384	0.152168	57.43697	52.16963	
38	56.75	57.76	0.5675	0.5776	0.164807	0.154451	57.15145	53.28601	
39	59.03	61.28	0.5903	0.6128	0.142177	0.122327	63.89889	60.52781	
40	61.63	63.42	0.6163	0.6342	0.119443	0.105495	71.00135	68.58123	

4-wash			R		K/S		removal		
	F	B	F	B	F	B	F	B	mean
21	50.39	51.98	0.5039	0.5198	0.24421	0.221808	32.373	27.26881	33.50
22	48.53	53.35	0.4853	0.5335	0.272941	0.203957	39.56657	38.00384	
23	50.46	53.32	0.5046	0.5332	0.243184	0.204334	33.76053	31.5729	
24	51.22	52.28	0.5122	0.5228	0.232281	0.217789	34.07182	31.56948	
25	50.78	54.91	0.5078	0.5491	0.23854	0.185131	33.09825	33.68997	
26	54.27	57.2	0.5427	0.572	0.192669	0.160126	50.91752	47.56454	48.17
27	55.42	58.4	0.5542	0.584	0.179301	0.148164	57.90705	51.99623	
28	56.13	57.98	0.5613	0.5798	0.171439	0.152266	52.94172	51.36693	
29	54.27	53.12	0.5427	0.5312	0.192669	0.206865	40.52928	40.70493	
30	53.66	56.83	0.5366	0.5683	0.200093	0.163967	45.66422	42.10444	
31	59.16	60.21	0.5916	0.6021	0.140966	0.131477	64.97975	59.48688	56.65
32	54.96	57.51	0.5496	0.5751	0.184553	0.156964	51.74344	46.41768	
33	57.12	56.73	0.5712	0.5673	0.16095	0.165018	50.74064	49.9513	
34	58.38	60.07	0.5838	0.6007	0.148358	0.132712	62.16649	61.42213	
35	57.69	59	0.5769	0.59	0.155151	0.142458	62.03868	57.54759	
36	62.13	64.13	0.6213	0.6413	0.115414	0.100316	71.84921	69.8575	65.61
37	57.11	59.81	0.5711	0.5981	0.161053	0.135031	59.68508	58.00331	
38	58.06	59.02	0.5806	0.5902	0.151478	0.14227	60.88081	57.27089	
39	60.47	62.27	0.6047	0.6227	0.129206	0.114305	67.48662	63.3891	
40	64.24	64.57	0.6424	0.6457	0.099531	0.097203	76.35864	71.35749	

[50°C]						
Before			R		K/S	
	F	B	F	B	F	B
1	46.02	50.24	0.4602	0.5024	0.316584	0.246423
2	45.3	49.05	0.453	0.4905	0.330253	0.264618
3	45.3	49.42	0.453	0.4942	0.330253	0.258836
4	45.43	50.77	0.4543	0.5077	0.327744	0.238684
5	42.78	46.29	0.4278	0.4629	0.38267	0.311597
6	45.86	49	0.4586	0.49	0.319575	0.265408
7	42.49	45.54	0.4249	0.4554	0.389197	0.325636
8	43.63	47.99	0.4363	0.4799	0.36415	0.281834
9	46.11	42.74	0.4611	0.4274	0.314913	0.383564
10	42.24	47.36	0.4224	0.4736	0.394912	0.292543
11	45.32	48.61	0.4532	0.4861	0.329866	0.271645
12	44.92	48.45	0.4492	0.4845	0.33769	0.274242
13	42.62	47.25	0.4262	0.4725	0.386258	0.294451
14	44.41	48.51	0.4441	0.4851	0.347923	0.273265
15	44.36	47.2	0.4436	0.472	0.348942	0.295322
16	42.86	44.79	0.4286	0.4479	0.380889	0.340271
17	43.18	47.76	0.4318	0.4776	0.373843	0.285701
18	43.94	46.22	0.4394	0.4622	0.357615	0.312883
19	44.22	47.61	0.4422	0.4761	0.35181	0.28825
20	43.31	47.13	0.4331	0.4713	0.371018	0.296545

1-wash			R		K/S		removal(%)		
	F	B	F	B	F	B	F	B	Average
1	52.76	55.96	0.5276	0.5596	0.211488	0.173295	34.46657	31.1497	30.26
2	49.99	53.02	0.4999	0.5302	0.25015	0.20814	25.14276	22.32699	
3	52.08	55.91	0.5208	0.5591	0.220461	0.173844	34.46146	34.38525	
4	52.98	56.49	0.5298	0.5649	0.208652	0.167562	37.67742	31.32782	
5	48.27	50.55	0.4827	0.5055	0.27719	0.24187	28.43066	23.24739	
6	56.81	58.68	0.5681	0.5868	0.164177	0.145479	50.46806	47.26326	39.96
7	51.65	55.81	0.5165	0.5581	0.226304	0.174947	43.14638	47.99402	
8	51.15	54.25	0.5115	0.5425	0.233267	0.192909	37.13116	32.91406	
9	53.44	50.93	0.5344	0.5093	0.202829	0.23639	36.96089	39.57339	
10	49.63	52.53	0.4963	0.5253	0.255605	0.214487	36.34876	27.78967	
11	52.3	54.09	0.523	0.5409	0.217523	0.194835	35.3052	29.54402	43.76
12	52.46	55.04	0.5246	0.5504	0.215407	0.18363	37.50676	34.50808	
13	52.61	55.51	0.5261	0.5551	0.21344	0.178289	46.13451	41.0773	
14	59.46	61.44	0.5946	0.6144	0.138201	0.121002	62.36848	58.20368	
15	54.46	56.72	0.5446	0.5672	0.190405	0.165123	47.00438	45.89945	
16	57.6	57.95	0.576	0.5795	0.156056	0.152563	60.89287	57.12187	52.95
17	60.51	61.72	0.6051	0.6172	0.12886	0.11871	67.64102	60.93673	
18	56.45	57.95	0.5645	0.5795	0.16799	0.152563	54.81239	53.22328	
19	54.36	55.84	0.5436	0.5584	0.191594	0.174615	47.10176	41.08418	
20	53.76	54.82	0.5376	0.5482	0.19886	0.186176	47.90736	38.74188	

2-wash			R		K/S		removal(%)		
	F	B	F	B	F	B	F	B	Average
1	54.11	57.68	0.5411	0.5768	0.194594	0.155252	40.007	38.83569	37.43
2	51.85	54.32	0.5185	0.5432	0.22357	0.192071	33.4857	28.67949	
3	52.85	57.27	0.5285	0.5727	0.210324	0.159407	37.64348	40.22604	
4	54.38	58.08	0.5438	0.5808	0.191356	0.151282	43.14961	38.4993	
5	50.86	53.06	0.5086	0.5306	0.237391	0.207629	39.15795	34.66325	
6	59.18	60.45	0.5918	0.6045	0.14078	0.12938	58.06653	53.60785	49.33
7	54.47	57.94	0.5447	0.5794	0.190286	0.152662	52.68659	55.09176	
8	54.26	56.8	0.5426	0.568	0.192789	0.164282	48.61462	43.50999	
9	56.84	53.3	0.5684	0.533	0.163862	0.204586	49.81043	48.1249	
10	52	54.98	0.52	0.5498	0.221538	0.184322	45.23763	38.52921	
11	55.47	56.93	0.5547	0.5693	0.178738	0.162922	47.49384	41.8193	53.55
12	55.06	57.25	0.5506	0.5725	0.1834	0.159612	47.32395	43.65491	
13	55.76	58.24	0.5576	0.5824	0.1755	0.149716	56.2626	51.18096	
14	62.63	63.38	0.6263	0.6338	0.111489	0.105792	70.3123	64.01769	
15	57.77	59.58	0.5777	0.5958	0.154351	0.137108	57.69397	55.77585	
16	62.11	61.51	0.6211	0.6151	0.115573	0.120426	71.85689	66.90151	62.78
17	64.03	63.97	0.6403	0.6397	0.101034	0.101466	75.32381	67.22916	
18	59.63	60.92	0.5963	0.6092	0.136654	0.125349	63.87009	62.25791	
19	57.79	58.79	0.5779	0.5879	0.154152	0.144435	58.1094	51.99596	
20	56.39	58.95	0.5639	0.5895	0.168632	0.142926	56.31892	53.9233	

3-wash			R		K/S		removal(%)		
	F	B	F	B	F	B	F	B	Average
1	55.58	58.63	0.5558	0.5863	0.177504	0.145956	45.61151	42.79539	42.30
2	52.74	54.91	0.5274	0.5491	0.211747	0.185131	37.19676	31.42317	
3	54.65	58.64	0.5465	0.5864	0.188163	0.14586	44.59932	45.70683	
4	56.16	59.32	0.5616	0.5932	0.171113	0.139486	49.55371	43.69504	
5	51.51	54.57	0.5151	0.5457	0.228235	0.189104	41.62568	40.8396	
6	60.72	62.33	0.6072	0.6233	0.127052	0.113832	62.52496	59.73518	52.87
7	57.03	59.03	0.5703	0.5903	0.161882	0.142177	60.21035	58.43112	
8	54.69	57.66	0.5469	0.5766	0.187694	0.155452	50.06012	46.77805	
9	53.57	55.45	0.5357	0.5545	0.201208	0.178963	37.49527	55.01461	
10	54.06	56.97	0.5406	0.5697	0.195198	0.162505	52.11047	46.29642	
11	57.1	58.51	0.571	0.5851	0.161157	0.147105	53.01903	47.90306	59.03
12	57.22	59.3	0.5722	0.593	0.15992	0.13967	54.52574	51.24958	
13	57.7	59.99	0.577	0.5999	0.155051	0.133422	61.72154	56.94292	
14	63.68	65.16	0.6368	0.6516	0.103576	0.093142	72.6657	68.85342	
15	59.61	61.12	0.5961	0.6112	0.136835	0.123663	62.88718	60.51565	
16	64.05	63.62	0.6405	0.6362	0.10089	0.104016	75.83363	71.89515	68.88
17	65.75	66.42	0.6575	0.6642	0.089206	0.084885	78.58949	73.27977	
18	61.91	62.62	0.6191	0.6262	0.117174	0.111567	69.50093	66.83313	
19	60.15	61.25	0.6015	0.6125	0.132005	0.122577	64.6202	59.89876	
20	60.08	61.56	0.6008	0.6156	0.132624	0.120016	66.33914	61.96541	

4-wash			R		K/S		removal(%)		
	F	B	F	B	F	B	F	B	Average
1	56.56	59.06	0.5656	0.5906	0.166817	0.141897	49.1164	44.5244	45.80
2	53.31	56.52	0.5331	0.5652	0.20446	0.167243	39.48391	38.49487	
3	55.85	59.31	0.5585	0.5931	0.174505	0.139578	48.88629	48.24841	
4	56.95	60.11	0.5695	0.6011	0.162713	0.132358	52.21133	46.83468	
5	52.54	55.62	0.5254	0.5562	0.214356	0.177057	45.36668	44.85617	
6	62.08	63.59	0.6208	0.6359	0.115812	0.104237	66.17519	63.51643	58.11
7	58.41	59.62	0.5841	0.5962	0.148068	0.136745	63.86927	60.16126	
8	56.76	59.42	0.5676	0.5942	0.164702	0.138568	56.58283	53.02767	
9	59.12	56.69	0.5912	0.5669	0.141337	0.16544	57.23815	58.6509	
10	55.27	56.92	0.5527	0.5692	0.181	0.163026	55.81519	46.11095	
11	58.28	59.5	0.5828	0.595	0.149327	0.137836	56.7366	51.46814	61.86
12	58.41	60.17	0.5841	0.6017	0.148068	0.131829	58.16117	54.23587	
13	58.56	61.09	0.5856	0.6109	0.146625	0.123915	63.97089	60.30502	
14	64.95	65.46	0.6495	0.6546	0.094573	0.091125	75.34306	69.62431	
15	60.6	62.06	0.606	0.6206	0.128083	0.115972	65.48233	63.22695	
16	65.01	65.38	0.6501	0.6538	0.094162	0.09166	77.65572	75.65539	71.90
17	67.1	67.83	0.671	0.6783	0.080656	0.076287	80.95012	76.41739	
18	63.48	64.07	0.6348	0.6407	0.10505	0.100746	73.00554	70.42534	
19	61.08	62.64	0.6108	0.6264	0.123999	0.111412	66.97406	63.93528	
20	61.48	62.4	0.6148	0.624	0.120673	0.113282	69.66483	64.32906	

[60°C]						
Before			R		K/S	
	F	B	F	B	F	B
21	46.89	49.53	0.4689	0.4953	0.300775	0.257139
22	43.4	49.05	0.434	0.4905	0.369074	0.264618
23	45.73	49.63	0.4573	0.4963	0.322024	0.255605
24	46.48	50.63	0.4648	0.5063	0.308131	0.240707
25	45.45	48.25	0.4545	0.4825	0.32736	0.277519
26	43.74	47.21	0.4374	0.4721	0.361818	0.295148
27	43.72	47.91	0.4372	0.4791	0.362241	0.283173
28	44.68	49.23	0.4468	0.4923	0.342469	0.261791
29	44.16	47.05	0.4416	0.4705	0.353046	0.297949
30	42.94	47.07	0.4294	0.4707	0.379115	0.297598
31	44.12	48.68	0.4412	0.4868	0.353873	0.270516
32	46.34	48.57	0.4634	0.4857	0.310681	0.272292
33	44.12	49.19	0.4412	0.4919	0.353873	0.262417
34	43.58	47.81	0.4358	0.4781	0.365215	0.284856
35	45.34	50.79	0.4534	0.5079	0.329479	0.238396
36	44.82	47.95	0.4482	0.4795	0.339673	0.282503
37	45.41	49.86	0.4541	0.4986	0.328129	0.252108
38	43.25	48.2	0.4325	0.482	0.372319	0.278344
39	44.47	47.97	0.4447	0.4797	0.346703	0.282168
40	41.19	44.8	0.4119	0.448	0.419837	0.340071

1-wash			R		K/S		removal(%)		
	F	B	F	B	F	B	F	B	Average
21	54.55	57.13	0.5455	0.5713	0.18934	0.160847	38.54364	39.22644	40.32
22	53.67	57.75	0.5367	0.5775	0.199969	0.154551	47.31355	43.51222	
23	56.21	58.09	0.5621	0.5809	0.170571	0.151183	48.79858	42.80566	
24	55.53	59.42	0.5553	0.5942	0.178064	0.138568	43.87195	44.59342	
25	51.22	52.67	0.5122	0.5267	0.232281	0.212657	30.11694	24.39737	
26	57.29	58.76	0.5729	0.5876	0.159203	0.144719	57.86422	53.06379	45.91
27	51.14	54.86	0.5114	0.5486	0.233408	0.185711	36.74854	35.8962	
28	55.85	58.46	0.5585	0.5846	0.174505	0.147586	50.77384	45.6584	
29	52.52	53.81	0.5252	0.5381	0.214618	0.198245	40.54895	34.82644	
30	55.57	57.43	0.5557	0.5743	0.177616	0.157775	54.83659	48.89987	
31	60.44	62.19	0.6044	0.6219	0.129467	0.114938	65.57525	60.10248	58.52
32	55.74	56.93	0.5574	0.5693	0.175722	0.162922	45.13392	41.96375	
33	59.63	61.61	0.5963	0.6161	0.136654	0.119607	63.47497	56.95195	
34	59.26	60.47	0.5926	0.6047	0.140039	0.129206	63.68921	56.97394	
35	63.03	64.42	0.6303	0.6442	0.108423	0.098256	69.5543	61.80764	
36	60.24	61.53	0.6024	0.6153	0.131213	0.120262	63.55255	59.90257	63.88
37	63.34	64.62	0.6334	0.6462	0.106091	0.096854	70.16146	64.56883	
38	62.26	63.13	0.6226	0.6313	0.114384	0.107666	71.518	64.00025	
39	62.23	63.25	0.6223	0.6325	0.114621	0.106764	69.26961	64.8428	
40	57.44	54.79	0.5744	0.5479	0.157674	0.186525	64.22806	46.75437	

2-wash			R		K/S		removal(%)		
	F	B	F	B	F	B	F	B	Average
21	54.98	58.07	0.5498	0.5807	0.184322	0.15138	40.27952	43.08305	46.92
22	53.85	59.45	0.5385	0.5945	0.197755	0.138293	47.93302	49.93939	
23	57.81	59.78	0.5781	0.5978	0.153952	0.1353	54.15333	49.31666	
24	58.59	61.54	0.5859	0.6154	0.146338	0.12018	54.57327	52.62144	
25	53.36	55.7	0.5336	0.557	0.203831	0.176166	39.1286	38.12308	
26	60.01	61.05	0.6001	0.6105	0.133244	0.124251	65.27752	60.28395	56.58
27	55.25	57.98	0.5525	0.5798	0.181227	0.152266	51.63269	48.21407	
28	59.05	61.13	0.5905	0.6113	0.14199	0.123579	60.60284	55.25608	
29	56.13	57.16	0.5613	0.5716	0.171439	0.160538	53.19713	47.9977	
30	59.3	60.24	0.593	0.6024	0.13967	0.131213	65.16325	58.18929	
31	63.71	65.11	0.6371	0.6511	0.103356	0.093481	73.20519	68.39153	67.34
32	59.48	59.58	0.5948	0.5958	0.138019	0.137108	57.74283	51.86809	
33	62.99	64.63	0.6299	0.6463	0.108727	0.096785	71.6358	66.05326	
34	62.46	64.04	0.6246	0.6404	0.112812	0.100962	71.39019	67.31243	
35	65.97	67.18	0.6597	0.6718	0.08777	0.080169	76.05261	69.78496	
36	64.08	64.52	0.6408	0.6452	0.100675	0.097554	72.86276	68.28689	73.93
37	67.16	67.99	0.6716	0.6799	0.080291	0.075352	78.31389	73.51135	
38	65.93	66.94	0.6593	0.6694	0.08803	0.081638	78.82513	73.76049	
39	66.82	67.08	0.6682	0.6708	0.082379	0.080779	78.89288	74.44891	
40	60.79	62.16	0.6079	0.6216	0.126454	0.115176	71.87669	68.48009	

3-wash			R		K/S		removal(%)		
	F	B	F	B	F	B	F	B	Average
21	57.9	60.04	0.579	0.6004	0.153058	0.132978	51.09315	50.5793	52.50
22	56.09	60.5	0.5609	0.605	0.171874	0.128946	55.17412	53.63434	
23	59.56	61.66	0.5956	0.6166	0.13729	0.119198	59.5221	55.91722	
24	59.52	61.75	0.5952	0.6175	0.137654	0.118467	57.50246	53.36937	
25	54.87	56.98	0.5487	0.5698	0.185595	0.162401	44.90522	43.30073	
26	61.55	62.56	0.6155	0.6256	0.120098	0.112033	69.03206	64.59388	61.65
27	57.63	59.59	0.5763	0.5959	0.155754	0.137017	58.89885	53.83053	
28	60.71	62.76	0.6071	0.6276	0.127138	0.110486	65.09261	60.49065	
29	57.56	58.59	0.5756	0.5859	0.156459	0.146338	57.58527	52.9576	
30	61.15	62.39	0.6115	0.6239	0.123411	0.11336	69.58797	64.43291	
31	65.09	66.46	0.6509	0.6646	0.093617	0.084632	76.05109	71.81003	71.28
32	60.89	61.76	0.6089	0.6176	0.125603	0.118385	61.89498	59.05155	
33	64.32	65.97	0.6432	0.6597	0.098963	0.08777	74.48889	69.6481	
34	64.49	65.74	0.6449	0.6574	0.097764	0.089272	75.64649	71.59145	
35	67.55	68.61	0.6755	0.6861	0.077942	0.071807	79.14489	73.47314	
36	66.89	66.96	0.6689	0.6696	0.081946	0.081514	78.57253	74.20882	78.74
37	69.45	70.18	0.6945	0.7018	0.067192	0.063354	82.45281	78.50147	
38	69.2	69.41	0.692	0.6941	0.068543	0.067407	84.22823	79.0965	
39	68.84	69.02	0.6884	0.6902	0.070522	0.069528	82.43185	78.60812	
40	63.49	63.75	0.6349	0.6375	0.104976	0.103064	77.13866	72.16818	

4-wash			R		K/S		removal(%)		
	F	B	F	B	F	B	F	B	Average
21	59.02	60.98	0.5902	0.6098	0.14227	0.124841	54.82434	53.89413	55.29
22	56.09	61.61	0.5609	0.6161	0.171874	0.119607	55.17412	57.32656	
23	59.73	62.36	0.5973	0.6236	0.13575	0.113596	60.01807	58.21383	
24	60.56	63.12	0.6056	0.6312	0.128427	0.107742	60.61451	58.05169	
25	55.69	57.92	0.5569	0.5792	0.176277	0.15286	47.85661	46.88956	
26	62.84	64.06	0.6284	0.6406	0.109872	0.100818	71.9525	68.54979	64.79
27	58.59	60.56	0.5859	0.6056	0.146338	0.128427	61.5846	56.9941	
28	62.08	63.59	0.6208	0.6359	0.115812	0.104237	68.5161	62.98881	
29	58.76	59.78	0.5876	0.5978	0.144719	0.1353	61.02414	56.8131	
30	61.86	63.85	0.6186	0.6385	0.117577	0.102335	71.17585	68.28871	
31	66.01	66.95	0.6601	0.6695	0.087511	0.081576	77.83541	72.99071	73.34
32	62.15	62.49	0.6215	0.6249	0.115255	0.112578	65.35551	61.27978	
33	65.93	66.96	0.6593	0.6696	0.08803	0.081514	77.68372	72.14288	
34	65.17	66.27	0.6517	0.6627	0.093074	0.085839	76.97297	72.84797	
35	68.65	69.3	0.6865	0.693	0.071582	0.068001	81.14614	75.15177	
36	67.95	68.06	0.6795	0.6806	0.075585	0.074946	80.5117	76.63406	81.26
37	71.25	72.06	0.7125	0.7206	0.058004	0.054166	85.35611	82.32247	
38	70.58	70.8	0.7058	0.708	0.061316	0.060215	86.23217	81.79356	
39	69.63	70.05	0.6963	0.7005	0.066231	0.064026	83.71247	80.64201	
40	65.07	65.31	0.6507	0.6531	0.093753	0.09213	79.88805	75.49761	
