# Supplementary material for: Kinetic analysis of hemoglobin detergency by probability density functional method
Source: PLoS One. 2020 Aug 7;15(8):e0237255. doi: 10.1371/journal.pone.0237255 (PMC7413519; doi:10.1371/journal.pone.0237255)
Supplement: Supplementary file 2 [file pone.0237255.s002.docx]

[20°C]						
Before			R		K/S	
	F	B	F	B	F	B
1	46.98	47.14	0.4698	0.4714	0.299183	0.29637
2	46.41	48.33	0.4641	0.4833	0.309404	0.276204
3	45.55	45.48	0.4555	0.4548	0.325445	0.326784
4	45.08	47.38	0.4508	0.4738	0.334539	0.292198
5	46.04	44.45	0.4604	0.4445	0.316212	0.347109
6	44.94	47.27	0.4494	0.4727	0.337295	0.294103
7	44.24	47.05	0.4424	0.4705	0.351399	0.297949
8	45.16	46.91	0.4516	0.4691	0.332974	0.300421
9	45.46	47.43	0.4546	0.4743	0.327168	0.291335
10	45.53	46.94	0.4553	0.4694	0.325827	0.29989
11	46.12	47.82	0.4612	0.4782	0.314728	0.284688
12	45.94	47.8	0.4594	0.478	0.318076	0.285025
13	45.48	47.62	0.4548	0.4762	0.326784	0.288079
14	44.43	47.25	0.4443	0.4725	0.347516	0.294451
15	47.26	47.8	0.4726	0.478	0.294277	0.285025
16	45.02	47.69	0.4502	0.4769	0.335718	0.286888
17	46.81	46.14	0.4681	0.4614	0.302198	0.314358
18	45.84	46.16	0.4584	0.4616	0.31995	0.313989
19	46.51	48.22	0.4651	0.4822	0.307588	0.278014
20	47.34	47.35	0.4734	0.4735	0.292889	0.292716

1-wash			R		K/S		Removal(%)		
	F	B	F	B	F	B	F	B	Average
1	47.52	47.07	0.4752	0.4707	0.289789	0.297598	3.267276	-0.4311	8.36
2	48.92	49.85	0.4892	0.4985	0.266677	0.252259	14.35036	9.051491	
3	46.52	47.11	0.4652	0.4711	0.307407	0.296896	5.748644	9.484723	
4	46.36	50.06	0.4636	0.5006	0.310316	0.249101	7.502319	15.36205	
5	47.64	46.17	0.4764	0.4617	0.287738	0.313804	9.349489	9.928535	
6	48.41	50.97	0.4841	0.5097	0.274894	0.235819	19.16269	20.63578	19.60
7	48.03	49.15	0.4803	0.4915	0.281166	0.263044	20.67268	12.19235	
8	48.66	51.09	0.4866	0.5109	0.270838	0.234115	19.33828	22.96225	
9	47.64	51.95	0.4764	0.5195	0.287738	0.222214	12.49729	24.71493	
10	50.23	50.25	0.5023	0.5025	0.246571	0.246275	25.22743	18.60147	
11	54.85	56.13	0.5485	0.5613	0.185827	0.171439	42.53226	41.47891	45.47
12	55.62	56.91	0.5562	0.5691	0.177057	0.16313	46.02221	44.5907	
13	54.55	56.42	0.5455	0.5642	0.18934	0.168311	43.61598	43.32877	
14	55.46	56.77	0.5546	0.5677	0.178851	0.164597	50.21968	45.91897	
15	57.97	57.5	0.5797	0.575	0.152365	0.157065	50.21374	46.80935	
16	55.38	58.08	0.5538	0.5808	0.179753	0.151282	48.12882	49.27077	49.95
17	58.01	58.96	0.5801	0.5896	0.15197	0.142833	51.70689	56.66582	
18	57.07	58.07	0.5707	0.5807	0.161467	0.15138	51.40737	53.78571	
19	57.35	59.06	0.5735	0.5906	0.15859	0.141897	50.34969	51.10417	
20	55.91	56.56	0.5591	0.5656	0.173844	0.166817	42.33036	44.79523	

2-wash			R		K/S		Removal(%)		
	F	B	F	B	F	B	F	B	Average
1	47.75	49.12	0.4775	0.4912	0.28587	0.263515	4.630003	11.53986	12.81
2	49.49	50.63	0.4949	0.5063	0.257755	0.240707	17.34682	13.41836	
3	47.96	46.57	0.4796	0.4657	0.282335	0.306503	13.73857	6.436143	
4	48.07	49.74	0.4807	0.4974	0.2805	0.253927	16.73682	13.64187	
5	48.26	47.68	0.4826	0.4768	0.277355	0.287058	12.75893	17.90192	
6	50.57	52.41	0.5057	0.5241	0.241578	0.216066	29.39381	27.62935	29.76
7	49.92	52.52	0.4992	0.5252	0.251203	0.214618	29.49226	29.10739	
8	52.88	55.4	0.5288	0.554	0.209937	0.179527	38.29204	41.86657	
9	49.33	53.8	0.4933	0.538	0.260232	0.198368	21.21539	33.24124	
10	50.63	50.58	0.5063	0.5058	0.240707	0.241433	27.09405	20.28134	
11	61.25	63.32	0.6125	0.6332	0.122577	0.10624	63.40239	65.35913	65.31
12	64.41	65.11	0.6441	0.6511	0.098327	0.093481	71.7162	70.0692	
13	64.43	64.24	0.6443	0.6424	0.098186	0.099531	72.54254	68.21114	
14	64.93	62.61	0.6493	0.6261	0.09471	0.111644	75.27237	64.64396	
15	59.37	57.55	0.5937	0.5755	0.139026	0.15656	54.93354	46.99427	
16	59.04	61.19	0.5904	0.6119	0.142083	0.123077	59.7532	59.51853	65.75
17	63.87	64.08	0.6387	0.6408	0.10219	0.100675	68.84078	70.59323	
18	63.07	63.51	0.6307	0.6351	0.10812	0.104828	68.71161	69.18364	
19	64.88	64.44	0.6488	0.6444	0.095054	0.098116	71.81991	67.54142	
20	61.54	61.1	0.6154	0.611	0.12018	0.123831	61.41264	60.08987	

3-wash			R		K/S		Removal(%)		
	F	B	F	B	F	B	F	B	Average
1	48.87	50.21	0.4887	0.5021	0.267473	0.246868	11.02878	17.38714	18.10
2	50.63	50.84	0.5063	0.5084	0.240707	0.237678	23.07267	14.56343	
3	48.69	49.12	0.4869	0.4912	0.270355	0.263515	17.55666	20.07756	
4	48.51	50.78	0.4851	0.5078	0.273265	0.23854	18.97744	19.12692	
5	49.01	48.61	0.4901	0.4861	0.26525	0.271645	16.73355	22.4966	
6	51.91	54.9	0.5191	0.549	0.222756	0.185247	35.17423	38.54117	39.12
7	51.19	53.69	0.5119	0.5369	0.232703	0.199722	34.93743	34.31059	
8	56.36	59.59	0.5636	0.5959	0.168954	0.137017	51.0469	56.58819	
9	52.09	55.36	0.5209	0.5536	0.220327	0.179979	33.86325	39.81634	
10	52.85	52.65	0.5285	0.5265	0.210324	0.212918	36.76505	30.17467	
11	66.89	67.2	0.6689	0.672	0.081946	0.080048	76.80887	74.95245	79.12
12	69.05	69.13	0.6905	0.6913	0.069363	0.068925	81.16875	79.05217	
13	68.34	68.35	0.6834	0.6835	0.073336	0.073279	80.42836	77.70849	
14	68.9	69.22	0.689	0.6922	0.070189	0.068435	82.57334	79.92382	
15	69.3	68.86	0.693	0.6886	0.068001	0.070411	80.06498	78.50859	
16	65.72	65.47	0.6572	0.6547	0.089403	0.091059	76.00966	71.152	79.67
17	71.03	71.05	0.7103	0.7105	0.059078	0.05898	83.6796	84.36769	
18	69.09	69.19	0.6909	0.6919	0.069144	0.068598	81.35435	81.16726	
19	69.11	70.82	0.6911	0.7082	0.069034	0.060115	80.61237	81.80832	
20	69	67.89	0.69	0.6789	0.069638	0.075935	79.38453	77.13106	

4-wash			R		K/S		Removal(%)		
	F	B	F	B	F	B	F	B	Average
1	49.4	50.57	0.494	0.5057	0.259146	0.241578	13.92485	19.24485	20.32
2	49.85	51.69	0.4985	0.5169	0.252259	0.225755	19.19273	19.07026	
3	49.82	49.7	0.4982	0.497	0.252713	0.254536	23.17897	22.92695	
4	48.91	51.7	0.4891	0.517	0.266836	0.225618	20.96874	23.73296	
5	49.49	48.46	0.4949	0.4846	0.257755	0.274079	19.1945	21.77105	
6	52.81	55.82	0.5281	0.5582	0.21084	0.174836	38.8333	42.22707	45.17
7	53.82	55.7	0.5382	0.557	0.198123	0.176166	45.11605	42.53869	
8	57.56	61.34	0.5756	0.6134	0.156459	0.121829	54.9357	61.84799	
9	54	58.4	0.54	0.584	0.195926	0.148164	41.59723	51.19202	
10	52.71	54.27	0.5271	0.5427	0.212137	0.192669	36.18803	37.19976	
11	70.64	68.93	0.7064	0.6893	0.061014	0.070024	83.71546	78.62391	83.45
12	70.93	70.74	0.7093	0.7074	0.05957	0.060514	84.36461	82.12913	
13	70.48	71.13	0.7048	0.7113	0.061821	0.058588	84.08244	83.02313	
14	71.7	71.42	0.717	0.7142	0.05585	0.057184	86.84285	83.90224	
15	71.13	72.05	0.7113	0.7205	0.058588	0.054213	83.39545	84.43418	
16	67.52	68.34	0.6752	0.6834	0.078121	0.073336	79.49117	77.59128	84.08
17	73.52	72.61	0.7352	0.7261	0.047687	0.05166	87.60023	86.78574	
18	71.05	70.95	0.7105	0.7095	0.05898	0.059472	84.65124	84.18589	
19	73.24	74.07	0.7324	0.7407	0.048887	0.045387	87.42057	87.33788	
20	71.43	70.36	0.7143	0.7036	0.057136	0.062431	83.82992	81.93597	

[35°C]						
Before			R		K/S	
	F	B	F	B	F	B
21	45.5	51.05	0.455	0.5105	0.326401	0.234682
22	44.72	50.14	0.4472	0.5014	0.341668	0.247908
23	45.73	51.09	0.4573	0.5109	0.322024	0.234115
24	47.06	49.11	0.4706	0.4911	0.297773	0.263673
25	45.27	50.59	0.4527	0.5059	0.330834	0.241288
26	44.59	48.84	0.4459	0.4884	0.344278	0.267951
27	43.46	49.63	0.4346	0.4963	0.367783	0.255605
28	43.9	49.13	0.439	0.4913	0.358452	0.263358
29	43.96	49.22	0.4396	0.4922	0.357198	0.261947
30	45.42	49.22	0.4542	0.4922	0.327937	0.261947
31	44.85	48.51	0.4485	0.4851	0.339077	0.273265
32	43.3	50.02	0.433	0.5002	0.371234	0.2497
33	43.89	50.02	0.4389	0.5002	0.358662	0.2497
34	43.81	49.33	0.4381	0.4933	0.360342	0.260232
35	44.58	48.74	0.4458	0.4874	0.344479	0.269551
36	44.63	48	0.4463	0.48	0.343473	0.281667
37	43.67	48.42	0.4367	0.4842	0.363301	0.274731
38	43.86	49.61	0.4386	0.4961	0.359291	0.255911
39	43.09	48.34	0.4309	0.4834	0.375812	0.27604
40	45.4	49.83	0.454	0.4983	0.328322	0.252562

1-wash			R		K/S		Removal(%)		
	F	B	F	B	F	B	F	B	Average
21	47.27	52.65	0.4727	0.5265	0.294103	0.212918	10.26173	9.758874	13.39
22	48.11	52.38	0.4811	0.5238	0.279835	0.216463	18.73689	13.31025	
23	48.03	52.97	0.4803	0.5297	0.281166	0.208781	13.16462	11.38868	
24	49.78	51.68	0.4978	0.5168	0.253319	0.225892	15.53726	14.99151	
25	49.65	51.08	0.4965	0.5108	0.255299	0.234257	23.6658	3.061895	
26	48.64	52.24	0.4864	0.5224	0.271161	0.218321	21.98241	19.36481	24.92
27	48.84	53.11	0.4884	0.5311	0.267951	0.206992	28.03314	19.92789	
28	48.6	53.8	0.486	0.538	0.271807	0.198368	24.98496	25.82077	
29	48.89	54.37	0.4889	0.5437	0.267154	0.191475	26.05908	28.15675	
30	51.31	53.57	0.5131	0.5357	0.231019	0.201208	30.64345	24.26783	
31	55.71	57.2	0.5571	0.572	0.176055	0.160126	49.79056	43.24834	50.83
32	58.3	59.3	0.583	0.593	0.149133	0.13967	61.76808	46.22345	
33	56.63	58.28	0.5663	0.5828	0.166074	0.149327	55.50062	42.16659	
34	58.41	59.48	0.5841	0.5948	0.148068	0.138019	60.87922	49.16639	
35	56.95	57.83	0.5695	0.5783	0.162713	0.153753	54.61425	44.90216	
36	59.84	59.53	0.5984	0.5953	0.134761	0.137563	62.90052	53.37077	57.98
37	59.54	59.83	0.5954	0.5983	0.137472	0.134851	64.22178	53.17216	
38	58.5	59.56	0.585	0.5956	0.147201	0.13729	61.01033	48.56568	
39	57.94	59.16	0.5794	0.5916	0.152662	0.140966	61.27964	51.09123	
40	62.05	62.21	0.6205	0.6221	0.116052	0.114779	67.0339	57.19471	

2-wash			R		K/S		Removal(%)		
	F	B	F	B	F	B	F	B	Average
21	48.63	53.72	0.4863	0.5372	0.271322	0.199352	17.49991	15.84152	15.27
22	48.56	52.17	0.4856	0.5217	0.272454	0.219255	20.97349	12.12826	
23	47.81	51.8	0.4781	0.518	0.284856	0.224251	11.9756	4.434238	
24	50.4	52.24	0.504	0.5224	0.244063	0.218321	18.77233	17.99585	
25	48.42	53.21	0.4842	0.5321	0.274731	0.205723	17.57763	15.48803	
26	50.2	54.03	0.502	0.5403	0.247016	0.195562	29.2414	28.24506	34.00
27	50.58	55.44	0.5058	0.5544	0.241433	0.179076	35.47946	31.37166	
28	50.61	55.64	0.5061	0.5564	0.240997	0.176834	33.86914	34.37628	
29	52.17	56.29	0.5217	0.5629	0.219255	0.169707	39.92124	36.85383	
30	53.44	54.99	0.5344	0.5499	0.202829	0.184206	39.55662	31.06086	
31	60.05	60.45	0.6005	0.6045	0.132889	0.12938	62.97424	55.00122	62.87
32	61.97	63.25	0.6197	0.6325	0.116692	0.106764	70.79014	60.04745	
33	61.67	62.71	0.6167	0.6271	0.119117	0.110871	69.03295	58.32203	
34	61.57	62.66	0.6157	0.6266	0.119934	0.111257	68.94792	59.93253	
35	60.24	62.08	0.6024	0.6208	0.131213	0.115812	64.07884	59.61415	
36	65.37	65.48	0.6537	0.6548	0.091727	0.090992	75.87013	70.61884	73.01
37	64.79	65.49	0.6479	0.6549	0.095674	0.090925	76.10825	69.86955	
38	65.15	65.82	0.6515	0.6582	0.09321	0.088748	76.54155	68.43959	
39	63.64	64.71	0.6364	0.6471	0.103869	0.096228	74.67857	68.01299	
40	67.44	66.99	0.6744	0.6699	0.0786	0.08133	78.86108	71.0798	

3-wash			R		K/S		Removal(%)		
	F	B	F	B	F	B	F	B	Average
21	48.91	54.25	0.4891	0.5425	0.266836	0.192909	18.92523	18.73052	19.01
22	48.32	52.56	0.4832	0.5256	0.276368	0.214094	19.7874	14.31303	
23	48.81	53.09	0.4881	0.5309	0.26843	0.207247	17.26814	12.07808	
24	50.08	53.22	0.5008	0.5322	0.248803	0.205596	17.11597	23.04505	
25	50.87	53.96	0.5087	0.5396	0.237248	0.196412	29.3216	19.54275	
26	49.74	55.02	0.4974	0.5502	0.253927	0.18386	27.16356	32.81073	38.65
27	52.21	56.93	0.5221	0.5693	0.218721	0.162922	41.85707	37.99381	
28	52.31	57.06	0.5231	0.5706	0.21739	0.161571	40.67637	40.44051	
29	52.7	56.72	0.527	0.5672	0.212267	0.165123	41.94377	38.68532	
30	55.06	56.85	0.5506	0.5685	0.1834	0.163757	45.69953	39.23101	
31	63.71	64.28	0.6371	0.6428	0.103356	0.099247	71.99435	66.51974	71.87
32	65.82	65.59	0.6582	0.6559	0.088748	0.090261	78.56171	66.98014	
33	64.1	66.24	0.641	0.6624	0.100531	0.086031	74.38908	68.75733	
34	65.8	66.61	0.658	0.6661	0.088878	0.083688	77.85446	71.02361	
35	65.04	64.78	0.6504	0.6478	0.093958	0.095743	75.27282	67.39622	
36	69.16	69.77	0.6916	0.6977	0.068761	0.06549	82.79141	80.06366	80.17
37	68.82	68.68	0.6882	0.6868	0.070633	0.071414	83.22948	77.28635	
38	68.34	68.44	0.6834	0.6844	0.073336	0.072767	82.25849	74.98231	
39	67.34	67.87	0.6734	0.6787	0.079201	0.076053	81.45286	75.6443	
40	70.84	70.53	0.7084	0.7053	0.060016	0.061568	84.72978	79.28314	

4-wash			R		K/S		Removal(%)		
	F	B	F	B	F	B	F	B	Average
21	49.59	55.2	0.4959	0.552	0.256218	0.181797	22.29882	23.71296	21.89
22	48.81	53.04	0.4881	0.5304	0.26843	0.207885	22.19279	16.94122	
23	50.26	53.65	0.5026	0.5365	0.246127	0.200216	24.45435	15.23851	
24	50.62	53.42	0.5062	0.5342	0.240852	0.203079	19.89484	24.04397	
25	50.18	54.81	0.5018	0.5481	0.247313	0.186292	26.16803	23.94991	
26	53.83	57.57	0.5383	0.5757	0.198	0.156358	43.97785	43.54176	47.17
27	54.12	58.78	0.5412	0.5878	0.194473	0.144529	48.666	45.53328	
28	53.69	59.61	0.5369	0.5961	0.199722	0.136835	45.7711	50.26787	
29	54.67	59.41	0.5467	0.5941	0.187928	0.138659	48.98738	49.25887	
30	56.25	58.49	0.5625	0.5849	0.170139	0.147297	49.89251	45.80768	
31	67.17	67.94	0.6717	0.6794	0.08023	0.075643	79.05761	75.54232	78.60
32	68.08	68.69	0.6808	0.6869	0.07483	0.071358	82.43226	74.92141	
33	68.41	69.28	0.6841	0.6928	0.072937	0.068109	82.3412	76.28631	
34	68.37	68.99	0.6837	0.6899	0.073165	0.069693	82.36102	76.65392	
35	68.11	68.01	0.6811	0.6801	0.074657	0.075236	81.07211	75.34811	
36	72.97	72.54	0.7297	0.7254	0.050063	0.051975	88.42658	85.0693	85.33
37	72.26	71.98	0.7226	0.7198	0.053246	0.054537	88.17409	83.70159	
38	71.36	71.67	0.7136	0.7167	0.057473	0.055992	86.82176	81.85025	
39	70.23	70.5	0.7023	0.705	0.063096	0.06172	85.87528	81.06556	
40	73.22	73.01	0.7322	0.7301	0.048974	0.049888	88.21692	84.13182	

[50°C]						
Before			R		K/S	
	F	B	F	B	F	B
1	45.86	49.36	0.4586	0.4936	0.319575	0.259766
2	43.99	51.37	0.4399	0.5137	0.356572	0.230181
3	44.63	52.14	0.4463	0.5214	0.343473	0.219657
4	46.39	50.34	0.4639	0.5034	0.309768	0.244946
5	46.49	49.11	0.4649	0.4911	0.30795	0.263673
6	43.67	48.97	0.4367	0.4897	0.363301	0.265883
7	43.79	50.61	0.4379	0.5061	0.360763	0.240997
8	44.79	52.4	0.4479	0.524	0.340271	0.216198
9	43.66	48.49	0.4366	0.4849	0.363513	0.27359
10	44.29	49.1	0.4429	0.491	0.350373	0.26383
11	45.41	49.51	0.4541	0.4951	0.328129	0.257447
12	45.61	48.72	0.4561	0.4872	0.324301	0.269873
13	44.42	47.7	0.4442	0.477	0.347719	0.286718
14	44.5	46.77	0.445	0.4677	0.346096	0.302911
15	46.1	47.9	0.461	0.479	0.315099	0.283341
16	46.97	47.83	0.4697	0.4783	0.299359	0.284519
17	45.82	46.21	0.4582	0.4621	0.320327	0.313067
18	45.3	47.39	0.453	0.4739	0.330253	0.292025
19	46.71	47.09	0.4671	0.4709	0.303985	0.297247
20	46.21	46.88	0.4621	0.4688	0.313067	0.300953

1-wash			R		K/S		Removal(%)		
	F	B	F	B	F	B	F	B	Average
1	52.61	54.55	0.5261	0.5455	0.21344	0.18934	34.46912	28.38546	27.20
2	50.98	56.15	0.5098	0.5615	0.235677	0.171222	35.05115	26.98101	
3	50.54	55.46	0.5054	0.5546	0.242015	0.178851	30.57678	19.61869	
4	50.17	54.9	0.5017	0.549	0.247462	0.185247	20.90085	25.59066	
5	51.89	52.96	0.5189	0.5296	0.223027	0.208909	28.66233	21.73069	
6	54.87	60.2	0.5487	0.602	0.185595	0.131565	50.53641	52.8351	48.31
7	54.86	58.74	0.5486	0.5874	0.185711	0.144909	50.1436	41.89852	
8	55.42	59.44	0.5542	0.5944	0.179301	0.138384	48.98497	38.04396	
9	56.75	57.31	0.5675	0.5731	0.164807	0.158998	56.47427	43.74935	
10	56.14	58.84	0.5614	0.5884	0.171331	0.143962	52.85981	47.53478	
11	71.7	71.58	0.717	0.7158	0.05585	0.056419	86.03685	81.78985	75.81
12	65.98	65.63	0.6598	0.6563	0.087705	0.089997	75.67672	69.66225	
13	66.06	65.95	0.6606	0.6595	0.087188	0.0879	77.52575	72.28248	
14	64.95	64.8	0.6495	0.648	0.094573	0.095605	75.20836	71.17813	
15	66.21	66.53	0.6621	0.6653	0.086223	0.084191	75.42764	73.30336	
16	73.8	74.09	0.738	0.7409	0.046507	0.045305	87.88814	87.66987	80.56
17	72.88	73.26	0.7288	0.7326	0.050459	0.048801	87.43037	87.6779	
18	66.28	66.51	0.6628	0.6651	0.085775	0.084317	76.73693	74.08528	
19	66.96	67.14	0.6696	0.6714	0.081514	0.080413	76.10411	75.92616	
20	66.42	67.19	0.6642	0.6719	0.084885	0.080108	75.70579	76.33974	

2-wash			R		K/S		Removal(%)		
	F	B	F	B	F	B	F	B	Average
1	54.25	56.71	0.5425	0.5671	0.192909	0.165229	41.13679	38.10376	37.49
2	52.47	58.71	0.5247	0.5871	0.215275	0.145194	40.9661	38.8922	
3	53.16	57.77	0.5316	0.5777	0.206357	0.154351	41.32342	31.39757	
4	53.48	56.36	0.5348	0.5636	0.202329	0.168954	36.04056	32.57475	
5	53.74	56.4	0.5374	0.564	0.199106	0.168525	36.73592	37.75534	
6	56.94	64.32	0.5694	0.6432	0.162817	0.098963	57.0139	65.65916	60.47
7	58.48	62.58	0.5848	0.6258	0.147393	0.111877	61.11968	56.30159	
8	59.65	63.74	0.5965	0.6374	0.136473	0.103137	62.0182	55.27681	
9	60.81	62.28	0.6081	0.6228	0.126283	0.114226	67.42322	60.84257	
10	58.6	62.12	0.586	0.6212	0.146242	0.115494	60.26677	58.82417	
11	76.95	76.85	0.7695	0.7685	0.034523	0.034868	92.77608	90.55808	86.84
12	72.34	71.9	0.7234	0.719	0.052881	0.05491	86.8157	83.2505	
13	72.19	72.17	0.7219	0.7217	0.053567	0.053659	87.53027	84.73135	
14	71.31	70.88	0.7131	0.7088	0.057714	0.059818	86.22966	83.46562	
15	73.05	72.77	0.7305	0.7277	0.049713	0.050946	87.45985	85.53993	
16	78.75	78.93	0.7875	0.7893	0.028671	0.028123	94.08774	93.967	90.27
17	78.36	77.54	0.7836	0.7754	0.029881	0.032528	94.09734	93.07668	
18	72.86	72.85	0.7286	0.7285	0.050548	0.050592	87.79427	86.11425	
19	73.81	73.63	0.7381	0.7363	0.046465	0.047221	88.09408	87.54844	
20	73.87	74.82	0.7387	0.7482	0.046215	0.042371	88.53586	89.38465	

3-wash			R		K/S		Removal(%)		
	F	B	F	B	F	B	F	B	Average
1	53.61	56.04	0.5361	0.5604	0.200712	0.17242	38.60269	35.20534	36.53
2	51.73	58.66	0.5173	0.5866	0.225207	0.14567	38.08661	38.67441	
3	52.72	58.24	0.5272	0.5824	0.212007	0.149716	39.62068	33.62581	
4	53.38	56.2	0.5338	0.562	0.20358	0.17068	35.62076	31.83501	
5	53.69	56.33	0.5369	0.5633	0.199722	0.169276	36.52786	37.45707	
6	60.1	63.57	0.601	0.6357	0.132447	0.104385	65.65075	63.52664	62.10
7	58.36	62.41	0.5836	0.6241	0.148551	0.113204	60.78796	55.72324	
8	60.18	63.94	0.6018	0.6394	0.131741	0.101683	63.45825	55.98752	
9	61.92	62.82	0.6192	0.6282	0.117094	0.110025	70.03501	62.44647	
10	60.33	61.99	0.6033	0.6199	0.130425	0.116532	64.9366	58.41256	
11	75.86	75.88	0.7586	0.7588	0.038409	0.038335	91.54805	89.14747	88.06
12	73.96	73.91	0.7396	0.7391	0.045841	0.046048	89.06731	86.68252	
13	73.66	72.64	0.7366	0.7264	0.047094	0.051526	89.4562	85.5067	
14	72.41	72.28	0.7241	0.7228	0.052562	0.053154	87.77004	85.75345	
15	73.83	73.73	0.7383	0.7373	0.046381	0.0468	88.55768	87.06611	
16	78.57	79.35	0.7857	0.7935	0.029225	0.02687	93.89498	94.4262	91.73
17	78.57	78.58	0.7857	0.7858	0.029225	0.029194	94.30969	94.18291	
18	74.62	74.81	0.7462	0.7481	0.043162	0.04241	90.11259	89.03258	
19	75.2	75.37	0.752	0.7537	0.040894	0.040244	89.99997	89.99153	
20	75.56	75.75	0.7556	0.7575	0.039526	0.038816	90.75512	90.61334	

4-wash			R		K/S		Removal(%)		
	F	B	F	B	F	B	F	B	Average
1	55.21	56.83	0.5521	0.5683	0.181683	0.163967	44.7826	38.6123	35.35
2	52.61	58.71	0.5261	0.5871	0.21344	0.145194	41.49836	38.8922	
3	52.6	56.63	0.526	0.5663	0.21357	0.166074	39.14943	25.76133	
4	50.75	55.17	0.5075	0.5517	0.238972	0.18214	23.74877	26.92258	
5	53.71	56.34	0.5371	0.5634	0.199475	0.169169	36.61114	37.49975	
6	62.33	64.32	0.6233	0.6432	0.113832	0.098963	70.94447	65.65916	65.93
7	59.48	64.03	0.5948	0.6403	0.138019	0.101034	63.80499	61.02974	
8	61.11	65.28	0.6111	0.6528	0.123747	0.092331	65.89096	60.55967	
9	63.43	64.09	0.6343	0.6409	0.105421	0.100603	73.3526	66.04361	
10	61.29	64.06	0.6129	0.6406	0.122244	0.100818	67.35202	64.64388	
11	77.85	77.83	0.7785	0.7783	0.031511	0.031576	93.72778	91.89757	90.21
12	74.83	75.1	0.7483	0.751	0.042331	0.041279	90.18997	88.52964	
13	74.92	74.38	0.7492	0.7438	0.041979	0.044124	90.97853	88.19788	
14	74.1	74.53	0.741	0.7453	0.045264	0.043521	89.95239	89.06109	
15	75.55	74.89	0.7555	0.7489	0.039563	0.042096	90.80464	88.7976	
16	79.53	80.52	0.7953	0.8052	0.026344	0.023564	94.8966	95.63782	93.47
17	79.87	80.23	0.7987	0.8023	0.025367	0.024358	95.55956	95.78737	
18	76.84	76.39	0.7684	0.7639	0.034903	0.036486	92.7049	91.14552	
19	76.94	76.61	0.7694	0.7661	0.034557	0.035706	92.16763	91.58041	
20	76.91	77.57	0.7691	0.7757	0.034661	0.032429	92.36931	92.82112	

[65°C]						
Before			R		K/S	
	F	B	F	B	F	B
21	45.16	49.06	0.4516	0.4906	0.332974	0.26446
22	46.67	48.66	0.4667	0.4866	0.304702	0.270838
23	43.94	50.31	0.4394	0.5031	0.357615	0.245388
24	45.45	50.21	0.4545	0.5021	0.32736	0.246868
25	43.35	49.75	0.4335	0.4975	0.370153	0.253775
26	43.84	50.86	0.4384	0.5086	0.359711	0.237391
27	44.59	48.68	0.4459	0.4868	0.344278	0.270516
28	45.96	50.22	0.4596	0.5022	0.317703	0.246719
29	44.63	49.01	0.4463	0.4901	0.343473	0.26525
30	44.38	52.91	0.4438	0.5291	0.348534	0.209551
31	44.4	50.62	0.444	0.5062	0.348126	0.240852
32	45.28	51.14	0.4528	0.5114	0.33064	0.233408
33	45.05	51.94	0.4505	0.5194	0.335128	0.222349
34	43.45	51.52	0.4345	0.5152	0.367998	0.228097
35	44.34	49.35	0.4434	0.4935	0.34935	0.259921
36	44.43	49.68	0.4443	0.4968	0.347516	0.254841
37	44.33	50.9	0.4433	0.509	0.349554	0.236818
38	46.18	50.78	0.4618	0.5078	0.31362	0.23854
39	45.95	49.85	0.4595	0.4985	0.317889	0.252259
40	45.68	50.61	0.4568	0.5061	0.322971	0.240997

1-wash			R		K/S		Removal(%)		
	F	B	F	B	F	B	F	B	Average
21	50.61	55.57	0.5061	0.5557	0.240997	0.177616	28.62547	34.35304	32.75
22	54.34	56.79	0.5434	0.5679	0.191832	0.164387	38.51666	41.07285	
23	50.39	52.52	0.5039	0.5252	0.24421	0.214618	32.78035	13.1649	
24	51.69	57.34	0.5169	0.5734	0.225755	0.158692	32.18413	37.48875	
25	51.07	55.56	0.5107	0.5556	0.234398	0.177728	37.86819	31.40964	
26	56.69	59.89	0.5669	0.5989	0.16544	0.134314	55.81707	45.66388	47.19
27	55.93	58.63	0.5593	0.5863	0.173625	0.145956	51.30625	48.11971	
28	55.98	58.24	0.5598	0.5824	0.173076	0.149716	47.25714	41.26758	
29	54.39	57.22	0.5439	0.5722	0.191237	0.15992	45.88028	41.5356	
30	56.07	60.94	0.5607	0.6094	0.172092	0.125179	52.37625	42.63575	
31	72.65	71.76	0.7265	0.7176	0.051481	0.055567	88.16517	80.84311	83.98
32	72.38	71.99	0.7238	0.7199	0.052699	0.054491	87.13477	80.68532	
33	70.92	71.18	0.7092	0.7118	0.05962	0.058345	85.17357	77.84244	
34	72.51	72.92	0.7251	0.7292	0.05211	0.050283	88.64869	82.15555	
35	71.7	71.7	0.717	0.717	0.05585	0.05585	86.91432	82.20056	
36	71.37	70.66	0.7137	0.7066	0.057424	0.060914	86.37408	79.74639	84.44
37	72.72	73.08	0.7272	0.7308	0.051169	0.049582	88.30767	83.15819	
38	72.95	72.84	0.7295	0.7284	0.050151	0.050636	87.25329	82.82127	
39	71.65	71.76	0.7165	0.7176	0.056087	0.055567	85.49267	81.75137	
40	73.13	72.11	0.7313	0.7211	0.049364	0.053935	87.88899	81.56681	

2-wash			R		K/S		Removal(%)		
	F	B	F	B	F	B	F	B	Average
21	51.51	56.29	0.5151	0.5629	0.228235	0.169707	32.59721	37.48154	36.77
22	55.32	57.69	0.5532	0.5769	0.180432	0.155151	42.40699	44.6362	
23	51.47	56.01	0.5147	0.5601	0.22879	0.172748	37.23779	31.07919	
24	52.51	56.49	0.5251	0.5649	0.21475	0.167562	35.67021	33.71726	
25	51.97	55.59	0.5197	0.5559	0.221944	0.177392	41.34243	31.54828	
26	58.12	62.19	0.5812	0.6219	0.150889	0.114938	59.99771	54.24766	55.63
27	58.23	60.92	0.5823	0.6092	0.149814	0.125349	58.46485	56.08063	
28	59.03	61.16	0.5903	0.6116	0.142177	0.123328	57.35355	52.49404	
29	56.72	60.03	0.5672	0.6003	0.165123	0.133067	53.75022	52.12497	
30	59.08	62.84	0.5908	0.6284	0.14171	0.109872	61.39521	50.37117	
31	75.56	74.85	0.7556	0.7485	0.039526	0.042253	91.71842	86.65239	89.36
32	75.49	75.51	0.7549	0.7551	0.039789	0.039714	91.1818	87.3492	
33	75.12	75.41	0.7512	0.7541	0.041202	0.040092	90.86752	86.50571	
34	75.7	75.82	0.757	0.7582	0.039002	0.038557	92.32726	87.57348	
35	74.88	75.33	0.7488	0.7533	0.042135	0.040396	90.97576	88.42544	
36	74.26	73.83	0.7426	0.7383	0.04461	0.046381	90.18956	85.72239	89.77
37	76.55	76.77	0.7655	0.7677	0.035918	0.035146	92.8212	89.56955	
38	76.44	76.33	0.7644	0.7633	0.036308	0.0367	91.83775	88.96358	
39	75.51	75.08	0.7551	0.7508	0.039714	0.041356	90.83926	87.65777	
40	76.1	75.93	0.761	0.7593	0.03753	0.038151	91.69026	88.44923	

3-wash			R		K/S		Removal(%)		
	F	B	F	B	F	B	F	B	Average
21	52.52	56.69	0.5252	0.5669	0.214618	0.16544	36.83514	39.16963	39.67
22	56.22	56.96	0.5622	0.5696	0.170463	0.162609	45.80891	41.75876	
23	52.2	55.74	0.522	0.5574	0.218854	0.175722	40.10963	29.8067	
24	54.05	57.94	0.5405	0.5794	0.195319	0.152662	41.82487	40.05243	
25	53.09	56.55	0.5309	0.5655	0.207247	0.166923	45.44199	35.87229	
26	60.87	63.53	0.6087	0.6353	0.125773	0.10468	67.21402	58.79205	60.87
27	60.49	62.11	0.6049	0.6211	0.129033	0.115573	64.71259	59.85695	
28	59.4	63.03	0.594	0.6303	0.138751	0.108423	58.47304	58.83492	
29	59.89	62.04	0.5989	0.6204	0.134314	0.116132	63.03541	58.80319	
30	60.62	63.65	0.6062	0.6365	0.12791	0.103796	65.49165	53.44137	
31	77.45	77.31	0.7745	0.7731	0.032828	0.033297	93.70912	90.55997	92.20
32	77.37	77.37	0.7737	0.7737	0.033095	0.033095	93.2804	90.33397	
33	76.85	77.09	0.7685	0.7709	0.034868	0.034043	92.82557	89.37701	
34	78.45	78.23	0.7845	0.7823	0.029599	0.030291	94.96615	91.39245	
35	77.02	78.07	0.7702	0.7807	0.034282	0.030801	93.30127	92.29046	
36	76.11	76.44	0.7611	0.7644	0.037494	0.036308	92.30836	89.86487	92.74
37	78.63	78.79	0.7863	0.7879	0.02904	0.028548	94.85683	92.49985	
38	78.02	78.59	0.7802	0.7859	0.030961	0.029163	93.60835	92.2857	
39	77.48	77.7	0.7748	0.777	0.032728	0.032001	93.1206	91.54628	
40	78.79	78.84	0.7879	0.7884	0.028548	0.028396	94.57546	92.70299	

4-wash			R		K/S		Removal(%)		
	F	B	F	B	F	B	F	B	Average
21	53.16	57.33	0.5316	0.5733	0.206357	0.158794	39.4063	41.79861	41.17
22	56.88	59.15	0.5688	0.5915	0.163444	0.141059	48.20435	50.07372	
23	52.62	57.06	0.5262	0.5706	0.213309	0.161571	41.71255	35.86133	
24	53.79	55.38	0.5379	0.5538	0.198491	0.179753	40.8203	28.53432	
25	53.3	57.3	0.533	0.573	0.204586	0.1591	46.18416	39.1034	
26	61.27	64.08	0.6127	0.6408	0.12241	0.100675	68.18016	60.56634	63.73
27	60.78	63.08	0.6078	0.6308	0.126539	0.108044	65.46237	62.76556	
28	61.71	63.56	0.6171	0.6356	0.118791	0.104458	64.99484	60.52163	
29	60.5	63.11	0.605	0.6311	0.128946	0.107817	64.65309	62.0818	
30	61.21	65.84	0.6121	0.6584	0.12291	0.088617	66.97598	61.11189	
31	77.39	78.31	0.7739	0.7831	0.033028	0.030038	93.64952	91.98184	93.49
32	79.22	78.82	0.7922	0.7882	0.027254	0.028457	95.11173	92.42579	
33	78.91	77.98	0.7891	0.7798	0.028183	0.03109	94.89219	90.77837	
34	79.4	79.28	0.794	0.7928	0.026723	0.027076	95.77317	92.87784	
35	78.6	78.26	0.786	0.7826	0.029132	0.030196	94.82626	92.53412	
36	78.09	78	0.7809	0.78	0.030737	0.031026	94.32026	92.03699	94.13
37	79.91	79.54	0.7991	0.7954	0.025254	0.026315	95.97721	93.49194	
38	79.66	80.21	0.7966	0.8021	0.025968	0.024414	95.26213	94.37915	
39	78.81	78.51	0.7881	0.7851	0.028487	0.029412	94.50538	92.62239	
40	79.54	79.38	0.7954	0.7938	0.026315	0.026782	95.293	93.40688	
