# Supplementary material for: Kinetic analysis of hemoglobin detergency by probability density functional method
Source: PLoS One. 2020 Aug 7;15(8):e0237255. doi: 10.1371/journal.pone.0237255 (PMC7413519; doi:10.1371/journal.pone.0237255)
Supplement: Attachment — Submitted filename: PLOSONE report.docx [file pone.0237255.s003.docx]

**Reviewers Comments and recommendation for Manuscript Number PONE-D-20-08400**

In this manuscript, the authors report washing tests performed on medical samples prepared by soiling of fabric substrate with haemoglobin. There is sufficient data provided for the method of washing of samples with special reference to the calculation of two parameters α_rl_ (Related to the cleaning mechanism) and µ_rl_ (Related to cleaning power).The process of cleaning has been studied as a function of pH, temperature and surfactant concentration and the data so obtained are explained on the basis of probability density functional method. The manuscript is well written and the results are explained properly.I recommend the publication of the manuscript in PLOSONE provided the following minor comments are addressed.

1. **In the abstract:** The first two lines in the beginning “Protein contamination----------theoron” should be deleted.

2 .**In the abstrac**t, the sentence starting from “ In this study------should be reworded and include washing tests performed by soiling of fabric and the continue as it is”

3**.In the introduction**: The lines 55 and 56 both starts both from “In this study..It should be corrected and reworded by deleting the words “in this” from line 55.

4. **Materials and methods:** Authors should mention the company and make of the thermostat/ water bath used for temperature control after pH in this section.

5.**In Fig4,5:** The authors should mention the values of regression coefficients(R^2^) either in the linear plots given in Fig 4 and Fig 5 or within the text at appropriate places.
