# Supplementary material for: Kinetic analysis of hemoglobin detergency by probability density functional method
Source: PLoS One. 2020 Aug 7;15(8):e0237255. doi: 10.1371/journal.pone.0237255 (PMC7413519; doi:10.1371/journal.pone.0237255)
Supplement: Attachment — Submitted filename: Response to Reviewers.docx [file pone.0237255.s004.docx]

Response to Reviewers

<To Editor>

Thank you for your valuable suggestions. It was corrected according to the instructions.

1) The following sentence was added to the introduction to explain the reason for determining the cleaning conditions.

(Revised Manuscript L66-70)

Since an alkaline solution is often used as a detergent for protein stains, it can be expected that the washing rate will change significantly depending on pH. Further, since SDS has a hemolytic action, it is expected that the concentration of SDS greatly contributes to the removability of hemoglobin stains. Moreover, since both can be regarded as a kind of chemical reaction, it can be predicted that they are affected by temperature.

2) The description of the statistical parameters was added as follows.

(Revised Manuscript L59-60)

the mean µ_rl_ and standard deviation σ_rl_ of the removal load distribution, assuming that the cleaning power follows a normal distribution.

<To Reviewer #1>

Thank you for your valuable suggestions about the selection of parameters. The manuscript was corrected as follows.

(Revised Manuscript L66-70)

Since an alkaline solution is often used as a detergent for protein stains, it can be expected that the washing rate will change significantly depending on pH. Further, since SDS has a hemolytic action, it is expected that the concentration of SDS greatly contributes to the removability of hemoglobin stains. Moreover, since both can be regarded as a kind of chemical reaction, it can be predicted that they are affected by temperature.

The structure of the manuscript has already been corrected by Editage, but if you point out more specific problems, I would like to ask Editage to respond again.

<To Reviewer #2>

We would like to thank you for your careful review and for your valuable suggestions.

1. In the abstract: The first two lines in the beginning “Protein contamination----------theoron” should be deleted.

The manuscript has been revised as instructed.

2 .In the abstract, the sentence starting from “ In this study------should be reworded and include washing tests performed by soiling of fabric and the continue as it is”

The manuscript has been revised as follows.

(Revised Manuscript L3-4)

In this study, washing tests were performed using samples prepared by contaminating fabrics with hemoglobin, and a kinetic analysis

3.In the introduction: The lines 55 and 56 both starts both from “In this study..It should be corrected and reworded by deleting the words “in this” from line 55.

The manuscript has been revised as instructed.

4. Materials and methods: Authors should mention the company and make of the thermostat/ water bath used for temperature control after pH in this section.

The following explanationwas added in L80-81

(temperature was controlled in the washing machine)

5.In Fig4,5: The authors should mention the values of regression coefficients(R2) either in the linear plots given in Fig 4 and Fig 5 or within the text at appropriate places.

The regression coefficients were added in Fig.4 and Fig. 7.

<To Reviewer #3>

Thank you for your valuable suggestions. The following modifications were made according to your instructions.

1) An explanation about the specifications of the fabric was added as follows.

(Revised Manuscript L78)

(weave density: 22.5/10 mm warp, 24.0/10mm weft, weight: 15.2 mg/cm^2^)

2) The following explanation was added about the reason why the test was performed at a temperature of 30°C or higher.

(Revised Manuscript L166-169)

Since it was expected that the cleaning efficiency would be low when the pH was low at low temperature and the cleaning effect due to the temperature and pH increase would be difficult to appear, the minimum temperature was set to 30 °C.
